# Combining established methods for value chain and power analyses: Towards a comprehensive next-generation understanding of value chain governance

**DOI:** 10.1016/j.mex.2025.103637

**Published:** 2025-09-19

**Authors:** Kendisha Soekardjo Hintz, La Thi Tham, Felister Mombo, Eckhard Auch, Jürgen Pretzsch, Lukas Giessen

**Affiliations:** a Chair of Tropical and International Forestry, Institute of International Forestry and Forest Products, Technische Universität Dresden, Germany; b Faculty of Political Economy, VNU University of Economics and Business, Hanoi, Vietnam; c Department of Forest and Environmental Economics, Sokoine University of Agriculture, Tanzania

**Keywords:** Actor-centered power, Institutional power, Power-over, Mixed-methods research, Natural resource-based value chain

## Abstract

Methods to analyze power in value chain governance often focus on multinational firms, which may not apply well to natural resource-based chains in domestic markets. Value chain analyses imply the complexity and power imbalances among the (in)direct actors in a value chain. However, quantifying the added value alone cannot fully capture the nuances of power among value chain actors. To cover the methodological gap, we propose complementing value chain analysis with actor-centered power and institutional power frameworks. The appropriation of profit and labor is framed as the key issue area to ensure coherence. Besides mapping the value chain and calculating the added value per value chain actor, the value network is mapped, and the power capabilities of the actors are quantified. The expected output identifies actors’ power in monetary and non-monetary terms, revealing how they utilize (in)formal institutional settings to their (dis)advantage. This multistage mixed-methods methodology permits the derivation of recommendations on how to achieve a more equitable value chain.

• The methodology consists of three stages: value chain analysis, actor-centered power and institutional power analyses, and synthesis
• The appropriation of profit and labor are the common issue area in both approaches
• The paper provides a step-by-step protocol for researchers to collect data

## Graphical abstract

**Figure:**
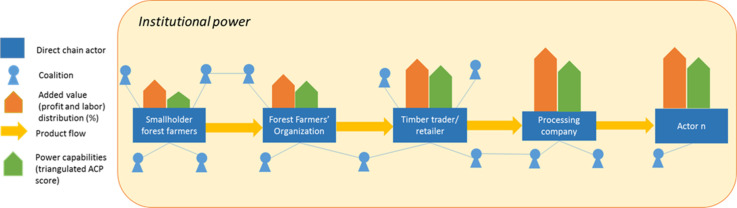

## Specifications table

**Table:** 

Subject area	Environmental Science
More specific subject area	*Value chain governance*
Name of your method	*Power in value chain governance*
Name and reference of original method	*Value chain*[1]*; Actor-centered power*[2]*;*
Resource availability	*Not available*

## Background

The evolution of value chain governance in the literature has been comprehensively studied, with a methodology that is heavily emphasized in the context of multinational firms or global value chains (see[3,4]). In a review of the concept of power in global value chains, Dallas et al. [3] concluded that empirical value chain governance studies have heavily relied on the classification of a) a producer- or a buyer-driven one[5] without considering the sources of power, or b) strong or weak bargaining power [6]. Regardless of the approaches, we identified a methodological gap, particularly in analyzing the governance of value chains for natural resource products associated with a network of actors with contrasting interests operating in a domestic market. Theories and methods for analyzing power have not been as well-integrated to explain the value chain governance of natural resource products. Thus, capturing the nuances of actors’ social relations and network, with an emphasis on local value chains, specifically in the context of contested natural resource products such as timber, is a lacuna. Ros-Tonen et al. [7] and Ingram[8] had, too, identified these gaps, thereby justifying the need to develop a methodology that integrates power approaches in analyzing value chain governance.

**The aim of this paper is to develop a methodology towards a comprehensive timber value chain analysis that integrates value chain analysis with the associated wider value network and the actors’ power capabilities.** The value chain concept has been instrumental in the forestry field, particularly in terms of analyzing how and at which stages value is added to the studied forest product and distributed among the different chain actors[9,10]. Here, we adopt a broad understanding of governance as aspects that comprise (in)formal regulatory structures, the interactions between actors, and the effects thereof on a value chain[11]. In developing a comprehensive value chain governance framework, we propose a twofold power analyses. The first layer refers to the actor-centered power (ACP) framework [2]. Inspired by Sahide et al. [12] and Zhao et al. [13], we advance the ACP framework by integrating it into the value chain scholarship. The ACP has been developed, applied, and proven helpful in explaining various forest governance contexts[2]. The second layer, institutional power, provides explanations for the extent to which actors utilize the (in)formal institutional settings to their advantage. As Shackleton et al. [14] argued, an actor-centered power analysis is complemented by institutional power analysis to achieve a more profound understanding. Furthermore, actors are defined as any [human] entity that has a particular interest in and the possibility of influencing the course of the studied value chain [15]. The ensuing ‘part 1′ addresses the scholarly gap by elaborating the theoretical underpinnings of both the value chain approach and power theories. ‘Part 2’ presents the step-by-step methods.

## Method details

### Part 1: power theories in value chain governance analyses

The search string “value chain governance” and “power” in the database Scopus yields 22 articles, published between 2003 and 2023. Among them, 12 articles were published in the last decade, which confirms the increasing trend in the topic. Of the 22 articles, 18 were considered for further scrutiny, as they provide comprehensive insights into the value chain governance of the studied products and power relations (see additional information 1 for an overview of the selected articles, including the concepts of power and value chain). Eight out of 18 selected articles shed light on value chain governance and power in the agricultural sector. Other fields included automotive (*n* = 3), energy (*n* = 2), information and technology (*n* = 2), and livestock and dairy (*n* = 2). This confirms that value chain governance, specifically in the context of power research, remains a gap in the literature, particularly in the forestry sector.

Among the selected articles, the works of Gereffi[16] and Gereffi et al. [6] dominated the concepts of value chain governance. Two studies[17,18] employed a value chain analysis, following the approach outlined by Kaplinsky and Morris[1]. Consequently, the concept of power in this school of thought refers to market share or market domination that centers on a lead firm. Another school of thought refers to the concept of power, as discussed by Dallas et al. [3], which was adopted by Zhong et al. [19] and Karlsen [20]. Additionally, Karlsen[20] employed Foucauldian discursive approach in exploring influential actors in the European offshore wind value chain governance. Among the selected studies, Purnomo et al. [17] employed the most similar approach to our proposed methodology. In studying palm oil value chain governance and power, Purnomo et al. [17] calculated the added value (i.e., the distribution of gross profit margin in their case) of each value chain actor, and overlaid it with social network analysis as well as ACP[2].

The selected articles shed light on the linkages between value chain analysis and power theories. Drawing from the selected articles in the agricultural sector, value chains involving small-scale suppliers have revealed that large buyers tend to exploit small-scale producers due to their position as lead firms[18,21]. Regardless of the type of value chain governance, power is treated as a unidimensional construct, whereas it is multidimensional and can be manifested in various activities within a value chain, such as margin, product and delivery-related activities[21]. This implies the need to strictly define the **issue area** to allow a coherent linkage between value chain analysis and power analysis. For our methodology, the issue area refers to the appropriation of net profit and labor to conduct timber-based business at the chain node.

Relevant for this methodology development is the work of Shackleton et al. [14] describing power theories in the context of (nature) conservation. According to their synthesis, the four most common concepts of power comprise **actors, institutions, structures, and discourses**. Although we acknowledge the typology of power in global value chains by Dallas et al. [3], the social theories of power[14] are particularly suitable for contextualizing value chain governance in the natural resources sector, such as the timber products sector. This is particularly relevant when the studied timber products stem from non-industrial, private, and small-scale tree farmers, who may be part of a collective, whose timber products are subjected to domestic value addition, but are then exported and further processed for sale as an end product abroad. The global value chains typology of Dallas et al. [3] has hitherto been used in the context of multinational companies, such as a grocery retail and petrochemical value chains[22], offshore wind in Europe[20], or green business in China[19]. Thus, we see the need for an alternative power analysis that departs from the multinational firm-centric view of Gereffi et al. [6] and Dallas et al. [3].

Exercising power can result in an outcome through three ways: power-to, power-over, and power-with[[23], [24], [25]]. Power-to refers to when individuals or groups (A and B) can use their capacity to support or resist an outcome. In contrast, power-over refers to when one party (A) may exert power over B to force or prevent an outcome. Power-with refers to when parties (A and B) collaborate in exercising power to support or resist an outcome[23,25]. Understanding these power manifestations is useful for choosing strategies for further actions, such as to understand and challenge power-over structures, to enhance power-to through capacity building efforts, or to enhance power-with by fostering cooperation[23]. Translating this framework into value chain governance, a lead firm has power over the weaker value chain actors. This justifies the need to scrutinize the power-over within a value chain governance. In this endeavor, we adopt several approaches to capture the power-over which implies multiple levels of analysis, as inspired by Dallas et al. [3].

What Dallas et al. [3] referred to as bargaining power, we argue that it can be captured through the ACP framework of Krott et al. [2], which is a manifestation of power-over. Bargaining power refers to the relationships between firms and can reveal different degrees of asymmetry in a hierarchy and market linkages[3]. The ACP was developed based on Weber’s definition of power, wherein power is defined as “*a social relationship where A (the potentate) changes the behavior of B (the subordinate) without recognizing B’s will*” (Krott 2014: 34). Altering a subordinate’s behavior can take the form of the potentate practicing coercion, using (dis-)incentives, and having dominant information (ibid). Coercion is observed when actor A alters the behavior of actor B by force, which may take the form of physical actions, threats, or bluffing about a threat[2]. Incentives are financial or non-financial factors that alter a subordinate’s behavior by motivation (ibid; [26]).

In line with the methodology of value chain analysis [1], the first point of entry is to map the value chain, which we interpret as mapping the network of value chain actors. A value chain network can be defined as any set of roles and interactions in which individuals or organizations engage in both tangible and intangible exchanges to achieve economic or social outcomes[27,4]. Value chain networks can be visualized using mapping tools, which can represent internal (i.e., interactions among chain actors) and external (i.e., interactions between people surrounding a direct chain actor) networks[28]. The latter is considered here as the coalition. The external network is considered saturated when the coalition actors do not mention any new actor. Understanding who the coalition members are behind a direct chain actor and the power dynamics among them allows for a better understanding and triangulation of the agency of the direct chain actor.

As Shackleton et al. [14] argued, the ACP is best done together with other approaches to achieve a more profound understanding, particularly to shed light on the power-over. Power types beyond actor-centered power contribute in shaping the power dynamics or (a)symmetries between actors[3]. Thus, the ACP is overlaid with the institutional power concept, which is a manifestation of power-over according to Partzsch [[29]; 2017]. According to Dallas et al. [3], institutional power in value chain governance operates through government regulation as well as collectives with some formal organization, such as business associations and multi-stakeholder initiatives. Charmakar et al. [30] complemented the notion of institutional power by delineating formal and informal institutions. Thus, institutional power is manifested in how power is exercised through institutional systems and public policies (i.e., formal institutions), as well as traditions, norms and customs (i.e., informal institutions) [30,14]. Thus, in the context of institutional power in timber value chains, an institution is seen as a process instead of a structure[31]. Institution as a process implies that, regardless its formality, institutions can be produced and reproduced, reflecting a constant dynamic that can constrain or enable human interactions (ibid). In effect, value chain actors are embedded in a certain institutional power that they can utilize to their advantage, thus leading to an intentional outcome, or possibly even an unintentional one. Analyzing the ACP and institutional power enables us to gain a more comprehensive understanding of the network associated with a value chain. The above-mentioned twofold power theories, including the relevant indicators, are summarized in Table 1 below.

**Table 1:** Research matrix: Variables of the twofold power analysis towards a comprehensive understanding of value chain governance.

Power	Variables	Indicators	Data collection	Data analysis
**Institutional Power**	Formal institution	Pathways of influence (how the following are enforced and who enforce them): Policy instruments, tax obligations and exemptions, licenses and permits, technologies	Sources of secondary data: International forest governance and policy arrangement[32]; policy or legal documents; institutional records Expert interviews, key informant interviews, group discussions, participant observation	Thematic analysis[33]
	Informal institution	Pathways of influence (how the following are enforced and who enforce them): Norms, customs, taboos		
**Actor-centered Power**	Coercion	Altering behavior by force, i.e., physical violence, threats of violence, monopoly of resources	Sequential design: quantitative network survey; qualitative semi-structured interviews, field observation, institutional records ([34,35], 2012)	Dominance degree calculation and qualitative triangulation ([36,35], 2016)
	(Dis-)incentives	Altering behavior by material (dis-)advantages, i.e., monetary transfers, financial contributions, in-kind contributions and services		
	Dominant information	Altering behavior by unverified information: Source of information, verification of information		

### Part 2: procedure of analyzing power in value chain governance analysis

Stage 1: Value chain analysis

The value chain framework[1] will be applied thoroughly to explore the production-to-consumption system of timber products as an illustrative case. The research will first map typical timber value chains, thereby highlighting main actors, their functions, and inter-relationships. The performance of a particular value chain and its actors will be examined through a set of indicators. The dataset will be managed in an Excel program applying the value chain expression presented by Tallec and Bockel [37], Klemperer [38], and Vedeld et al. [39].

Value chain analysis helps to understand how value is created and distributed across the different stages of product or service transformation. According to Tallec and Bockel [37] and Klemperer [38], added value is the difference between the revenues from total sales and the cost of externally supplied inputs. Alternatively, this can be presented as the contribution of a firm to employment, government and its investors[39]. Thus, the added value in our proposed methodology can be calculated as follows:

VAi=Labourcosts+taxes+netprofit

where VAi represents the added value at ith stage. The added value for the entire value chain is determined by the summation of values generated at each stage of the chain. To make the value created comparable across the different chains, we calculate the added value per a certain unit of the product, e.g., per m^3^ [40]. However, for the purpose of our research, which focuses on timber products from small-scale producers, we consider the appropriation of net profit and labor as the key issue area for the value chain governance and power study. We deliberately exclude the contribution to the government (i.e., taxes) because, based on empirical studies and our own experiences, small-scale producers are not always subject to taxation. The expected output of this step is to quantify the added value in monetary terms for each timber value chain actor and its corresponding added value distribution (in percentage). Examples of this stage in the natural resources sector refer to the publications by Tham et al. [10] and Temu et al. [9].

Stage 2: Multilevel power analysis in value chain governance

Step 2.1: Actor-centered Power (ACP) approach: Mapping actors in the wider network

Complementing the value chain analysis approach, the power relations among the actors within the value chain governance are analyzed as the next step. At this stage, the ACP analysis aims to identify which value chain actors are the most powerful in terms of the issue area, specifically the appropriation of net profit and labor. The first step of the ACP is to identify the actors. As the direct value chain actors have already been identified in the previous stage, other actors in the wider value network need to be identified to capture the entire network. Hence, we have two units of analysis: the direct chain actors, and the indirect actors surrounding each direct chain actor. This network analysis can be done using network analysis tools, such as Gephi or UCINET.

The starting point is each of the direct chain actor. Through snowball or purposive sampling, the other actors surrounding a direct chain actor are identified, so as to ascertain whether a coalition is formed behind each direct value chain actor. Actors in the wider value network (henceforth coalition members) should have a particular interest and the possibility of influencing the course of the chain[15]. Thus, actors are not only limited to individual persons, but can also include a collective of individuals that represent a converging interest[13]. A coalition member may be linked to more than one direct value chain actors. After identifying coalition members, the wider value network is mapped, as shown in Fig. 2.

Considering the exploratory nature of the research, the anticipation of identifying informal actors, and the lack of an established population size, the use of snowball or purposive sampling as a non-probability sampling technique is justified to identify actors involved in the value chain network. The use of a probability sampling technique can be considered if the population size was known, such as in the form of a database of all relevant actors in each stage of the value chain. However, based on past experiences, such a database rarely exists or is costly to establish or access, and the source of the database also needs to be considered for its reliability.

Step 2.2: Actor-centered power (ACP) approach: Dominance degree

The ACP specifies three elements of power: coercion, (dis)incentives, and dominant information[2]. The power elements of each actors are ascertained using the sequence design methodology, which first begins with a quantitative network survey and is followed by qualitative in-depth interviews[36]. The quantitative network survey has twofold aims, namely to identify the actors as well as their power elements in terms of dominance degree (ibid). Considering the issue areas of appropriation of net profit and labor, we propose the following standardized and generic quantitative network survey questions adapted from Schusser[36]:

1 List all the actors you deal with in relation to your timber-related business.
2 Who of these actors provides you with information related to your timber-related business (e.g., your ability to be profitable and to access or employ labor)? How good was this information according to your own judgment? (0 no or unacceptable information, 1 acceptable/good information, 2 very good information)
3 Have you ever verified this information? (0 always, 1 never, 2 sometimes)
4 Who of these actors provides incentives (e.g., money, financial contribution, in-kind contribution, subsidy) (0 no incentives, 1 material or moral incentives or disincentives)
5 Apart from the information or provided incentives, is one of these actors still needed to carry out your activities related to your timber-related business? (0 not needed, 1 needed)
6 Do you need to get permission from one of your mentioned actors to carry out your activities related to your timber-related business? (0 not needed, 1 needed)

The quantitative dataset can be managed in an Excel program to calculate the dominance degree of the identified actors (see[35]). The dominance degree is calculated with the following formula:

$$h_{i}=\frac{X_{i}}{\sum_{i}^{n}X_{i}}\;\;\;\;\;\;CR_{m}=\sum_{j=1}^{m}h_{r}\;\;\;\;\;\;D_{m}=\frac{{\left(CR_{m}\right)}^{2}}{m}+\frac{{\left(1-CR_{m}\right)}^{2}}{n-m}$$

where n, total number of identified actors

X_i_, Sum of answers per actor for one power element

H_i_, the ratio of power per actor and per one power element (i) r, the position of the sorted ratio of power per actor (h_i_), the sorting starts with the highest h_i_ value until the lowest, whereas equal values can be sorted continually m, number of considered powerful actors

CR_m_, concentration ratio, which shows the distribution of power per actor (i.e., CR_2_=0.4 means that the first two actors hold 40 % of the total available power per element in the network)

D_m_, dominance degree, with *m*=group of powerful actors and n-m group of less powerful actor ([35], p. 183).

Based on the scores, the dominance degree of each actor will be calculated, thereby classifying the actors into two groups: those with greater and those with lesser power. Focusing only on the powerful actors (i.e., those with the higher dominance degree), the follow-up qualitative in-depth interviews in the ACP method helps to confirm the power features, which are to be complemented by empirical observation and secondary data such as financial records or institutional records. Through the qualitative method, the researcher can observe the power resources of each of the powerful actors. If an observation is empirically found, then it corroborates the quantitative dominance degree and confers the actor with a score of 2 (more powerful group). If no power element is observed or no additional data, then it does not corroborate the dominance degree and confers the actor with a score of 1 (less powerful group). The triangulated ACP score (1 or 2) complements the added value distribution calculated previously, as demonstrated in the following Fig. 3. A publication employing the ACP method in the field of natural resource value chains includes Purnomo et al. [17].

Step 2.3: Institutional power

Analyzing institutional power follows a qualitative data collection and analysis strategies. Studies on institutional power in the context of value chain analyses have employed qualitative research strategies [41,42,22]. We propose that institutional power, both formal and informal, in a chain can be ascertained through key informant and expert interviews as well as participant observation and secondary data of relevant documents. The actors identified in the wider network of the chain (step 2.1) may offer further insights to the decision-making within the node, and can be considered as key informants or experts.

The recommended steps in analyzing institutional power are as follows. The starting point is **formal institutional power**, which also reveals the political and legal dimensions of a structural power. Relevant for timber-related chain would be any forest governance and policy instruments that regulate land tenure, tree tenure, harvesting permits and licenses, taxation, timber trade and export of the supplier country, market trends, and technology adoption. As a point of departure, the overview of forest governance and policy instruments[32] can be used as a starting point to identify relevant policy instruments. Within the studied timber supplier country, legal documents associated with forest land use, timber trade, and taxation, among others, should be considered and compiled.

Parallel to the formal institutional power, **informal institutional power** can be manifested through the norms, customs, and taboos that are relevant to the actors in the chain and in the wider network[30]. Insights regarding informal institutional power are usually held by the so-called endogenous actors, such as village leaders or elders (ibid). The qualitative data can be analyzed by thematic analysis, specifically deductive coding of the qualitative data, following the variables under formal and informal institutional power (see Table 1). Emerging themes can be coded inductively. The thematic analysis can be performed using qualitative coding software programs, such as NVivo, MaxQDA, or Atlas.ti. As elaborated in the preceding ‘Part 1′ of this paper, the formal and informal institutional power analysis had not been done in a value chain governance study. However, the framework had been employed in another context, e.g., community forestry governance [[30], [43]].

Stage 3: Synthesizing two approaches for analyzing power in value chain governance

**Fig. 1:**
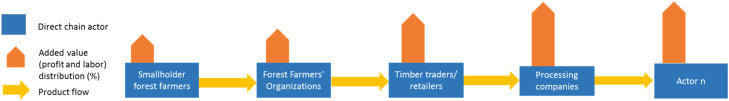
Value chain analysis (simplified and for illustrative purposes, arrows are not for scale).

The calculation of the value added (quantitative data) will be complemented with the value chain network and power analyses (quantitative and qualitative data), and embedded within an understanding of the institutional power (qualitative data). Overall, steps above build up on each other. Consequently, the expected outputs of the above-mentioned methods comprise:

1 The map of actors in the wider network.
2 Quantification of the added value per direct value chain actors (in percentage, see Fig. 1).

**Fig. 2:**
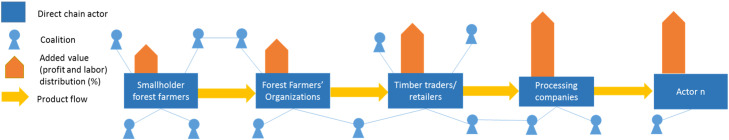
Actors mapping in the wider value network (simplified and for illustrative purposes, arrows are not for scale).
3 Quantification of power capabilities of direct value chain actors and coalition members (in dominance degree and the triangulated ACP score, see Fig. 3).

**Fig. 3:**
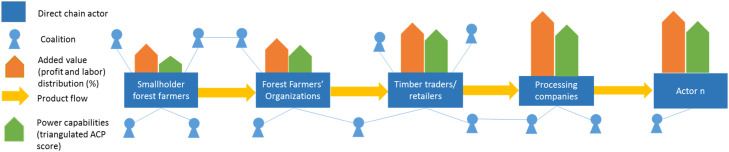
A sketch of the wider value network, with the added value distribution and actor’s triangulated ACP score (simplified and for illustrative purposes, arrows are not for scale).
4 Discussion on power asymmetries by using the ratio of added value distribution (i.e., net profit and labor costs, expressed as a percentage) to power capabilities (ACP triangulated score) of direct value chain actors, relative to one another. Focusing on the direct value chain actors, there are four scenarios to be expected: higher added value, higher triangulated ACP score (2); higher added value, lower triangulated score (1); lower added value, higher triangulated score (2); and lower added value, lower triangulated score (1). The ratios enable nuanced discussions to assess the monetary and non-monetary power of direct value chain actors, in terms of their ability to appropriate profit and access to labor. To explain each of the possible scenarios, the power features of the coalition members surrounding each of the direct value chain actor can be considered. The question to ask here is “to what extent is the direct value chain actor influenced by the coalition members?”
5 Discussion on the formal and informal institutional power regarding to what extent do the more and less powerful actors (as analyzed in the previous steps) make use of the institutional power arrangements to their (dis)advantage (see Fig. 4). Here, the questions to ask are “which formal and informal institutional power arrangements are observed? How did this lead to the actor’s (dis)advantage?” Following, the explanation on the power manifestations to the questions above can be explained by the power-over, power-to, and power-with framework of Partzsch [[25], [29]] (see the sub-chapter ‘Method Details’ above).

**Fig. 4:**
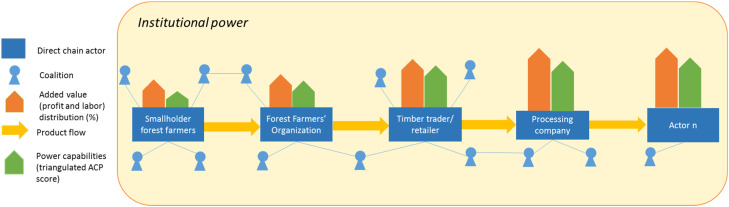
Comprehensive value chain governance analysis (simplified and for illustrative purposes, arrows are not for scale).

## Limitations

Following, we reflect on some limitations of the proposed methodology, while proposing some solutions. The lack of practical examples or simulated datasets here may be a potential challenge. Nonetheless, the modular steps are proven and tested methodologies. The novelty lies in the integration of the different schools of thought (i.e., value chain analysis and the twofold power frameworks) and offering an alternative to Gereffi’s [16] legacy on the global value chain governance framework. Therefore, our proposal needs to be tested with empirical analyses in various timber and non-timber forest product value chains, thereby expanding to other natural resource-based value chain contexts. We acknowledge that there may be other power sources, such as the power geography (i.e., the remoteness of a landscape) [44], and structural and discursive power[14] or all of the four dimensions of power-over[25], which could further explain the governance of natural resource-based value chains. However, the scope in our proposed methodology is justified because the twofold power frameworks specifically cover the prevailing methodological gap, as identified in ‘Part 1′ of this manuscript.

Implementing the methodology in the field implies challenges, notably the time-intensive nature of the data collection and analysis procedures. Although the mixed-methods approach is intended to minimize bias, the steps concerning power analyses rely on self-reported data or observations, which are made in a cross-sectional manner, and can be affected by bias and misinterpretation. Drawing on experiences, researchers can anticipate a two-stage field trip, where the first stage focuses on value chain analysis and actor mapping. After gaining clarity on the value chain and the network, researchers can return to undertake the second stage, focusing on the power frameworks. The time in between allows some time for reflection and further planning.

The second stage of the data collection may be influenced by political sensitivity, thus exposing participants in vulnerable positions to risks. In this case, researchers should prioritize research ethics and the safety of both interviewees and themselves[45]. The free, prior, and informed consent principle shall be applied, ensuring anonymity and confidentiality, and adhering to the interviewees’ cultural norms and practices (see[45]). Furthermore, the data collection methods in disruptive situations can be adapted to the specific situation while remaining focused on the goal, such as using remote data collection tools or collaborating with local actors through participatory research (see[46]). On this note, we recommend holding a multi-stakeholder workshop with the identified actors from the wider value chain. For instance, the Participative Innovation Platform (PIP) [47] has been developed and tested in the context of (timber) value chain analysis in tropical countries (see[48,9]). Thus, we recommend holding a PIP workshop to bring together actors in the chain network and discuss challenges and the way forward, notably in terms of strategies to empower less powerful actors in the value chain, i.e., to harness the power-to and power-with [[25], [29]] of these entities.

In the long run, researchers employing this methodology can share case-specific datasets as supplemental material in future studies. Such a collective action approach permits the exchange of lessons learned, thereby enhancing the methodology’s reproducibility and overall applicability. Ultimately, issues of political sensitivity, confrontation, and handling in the field should be addressed in publications. Equally, researchers should continually reflect on their positionality, personal biases, and influence on the study’s outcomes. While measuring these influences be elusive, reflecting on the researcher’s role is essential and can follow the well-tested analytical framework of, for example, Cunliffe and Karunanayake[49].

## Ethics statements

We affirm that the work presented in this manuscript did not involve human participants, animal experimentation, or the use of data sourced from social media platforms. However, if the methodology described in this paper is implemented in future studies involving human subjects, appropriate informed consent will be obtained from all participants.

## CRediT author statement

KSH: Conceptualization; Methodology; Writing – Original draft; Writing – Review & editing; Funding acquisition. LTT: Writing – Review & editing; Funding acquisition. FM: Writing – Review & editing; Funding acquisition. EA: Conceptualization; Methodology; Writing – Review & editing. JP: Conceptualization; Methodology; Writing – Review & editing. LG: Conceptualization; Methodology; Writing – Review & editing; Funding acquisition.

## Declaration of competing interest

The authors declare that they have no known competing financial interests or personal relationships that could have appeared to influence the work reported in this paper.

## Data Availability

No data was used for the research described in the article.

## Appendix Supplementary materials

Image, application 1
