# Supplementary material for: Combining established methods for value chain and power analyses: Towards a comprehensive next-generation understanding of value chain governance
Source: MethodsX. 2025 Sep 19;15:103637. doi: 10.1016/j.mex.2025.103637 (PMC12493210; doi:10.1016/j.mex.2025.103637)
Supplement: Supplementary file 1 [file mmc1.docx]

**Additional information**

**Additional information 1**

An overview of selected articles (n = 18) on “value chain governance” and “power” from Scopus database

| **Sector** | **Type of product** | **Geographical context** | **Concept of value chain** | **Concept of power** | **Sources** |
| --- | --- | --- | --- | --- | --- |
| Agriculture | Global fresh fruit and vegetable chain | South Africa – European Union | Global value chains (Gereffi 1994) | Market share; buyer power | Gibbon 2003 |
|  | Food | Global | Supply chain and value chain governance | Market power | Sodano et al. 2008 |
|  | Maize, sugar cane, sunflower oil | Uganda | Value chain governance (Gereffi et al. 2005) | Market share | Johnston and Meyer 2008 |
|  | Oil palm | Indonesia | Value chain analysis (Kaplinsky and Morris 2001; Gereffi et al. 2005) | Actor-centred power (Krott et al. 2014) | Purnomo et al. 2018 |
|  | Medicinal plants | Albania | Supply chain | Market power; negotiation power | Xhoxhi et al. 2020 |
|  | Maize seeds | Ghana | Value chain analysis (Gereffi 1994; Kaplinsky and Morris 2001) | Power to influence; power asymmetry; power concentration | Quarshie et al. 2021 |
|  | Commercial agriculture | Sri Lanka | Supply chain; Value chain governance | Power asymmetry | Senevirathna 2023 |
|  | Palm oil | Colombia | Global value chains (Gereffi 2005) | Workers’ power; i.e., structural power and associational power | Serrano 2023 |
| Livestock and dairy | Pig | Myanmar | Value chain governance (Gereffi et al. 2005) | Powerful actors are those who control transactions along the value chain (lead firms) (Mccormick and Schmitz 2001) | Ebata et al. 2020 |
|  | Milk | Vietnam | Value chain governance (Gereffi et al. 2005) | Supplier power and buyer power | Hoang et al. 2021 |
| Automotive | Automotive | Global | Global value chain (Gereffi 2005) | Market share concentration; Coordination and buying power | Sturgeon et al. 2009 |
|  | Modular components | Turkey | Global value chain (Gereffi 2005) | Power asymmetry of lead firms | Özataǧan 2011a |
|  | Modular components | Turkey | Global value chain (Gereffi 2005) | Power asymmetry of lead firms | Özataǧan 2011b |
| Energy | Offshore wind | Europe | Global value chain (Ponte and Gibbon 2005) | Power relations through discursive approach | Karlsen 2018 |
|  | Offshore wind | Europe | Global value chain | Lead firms | van der Loos et al. 2022 |
| Information and Communications Technology | Mobile communication | Global | Global value chain | Power relations: monopoly power, bargaining power | Wen and Yang 2010 |
|  | IT service | South Africa | Global value chain (Gereffi 1994; Gereffi et al. 2005); value chain upgrading | Power balance between lead firms and producers | Keijser et al. 2021 |
| Other | Green events | China | Value chain governance (Gereffi 1994; Gereffi et al. 2005) | Power in global value chains (Ponte et al. 2019) | Zhong et al. 2021 |

Articles from Scopus:

Ebata, A.; MacGregor, H.; Loevinsohn, M.; Win, K. S.; Tucker, A. W. (2020): Value Chain Governance, Power and Negative Externalities: What Influences Efforts to Control Pig Diseases in Myanmar? In *Eur J Dev Res* 32 (3), pp. 759–780. DOI: 10.1057/s41287-019-00239-x.

Gibbon, P. (2003): Value‐chain Governance, Public Regulation and Entry Barriers in the Global Fresh Fruit and Vegetable Chain into the EU. In *Development Policy Review* 21 (5-6), pp. 615–625. DOI: 10.1111/j.1467-8659.2003.00227.x.

Hoang, V.; Nguyen, A.; Hubbard, C.; Nguyen, K-D. (2021): Exploring the Governance and Fairness in the Milk Value Chain: A Case Study in Vietnam. In *Agriculture* 11 (9), p. 884. DOI: 10.3390/agriculture11090884.

Johnston, C.; Meyer, R. (2008): Value chain governance and access to finance: Maize, sugar cane and sunflower oil in Uganda. In *Enterprise Development & Microfinance* 19 (4), pp. 281–300. DOI: 10.3362/1755-1986.2008.026.

Karlsen, A. (2018): Framing industrialization of the offshore wind value chain – A discourse approach to an event. In *Geoforum* 88 (6), pp. 148–156. DOI: 10.1016/j.geoforum.2017.11.018.

Keijser, C.; Belderbos, R.; Goedhuys, M. (2021): Governance and learning in global, regional, and local value chains: The IT enabled services industry in South Africa. In *World Development* 141 (4), p. 105398. DOI: 10.1016/j.worlddev.2021.105398.

Özataǧan, G. (2011a): Shifts in Value Chain Governance and Upgrading in the European Periphery of Automotive Production: Evidence from Bursa, Turkey. In *Environ Plan A* 43 (4), pp. 885–903. DOI: 10.1068/a43428.

Özatağan, G. (2011b): Dynamics of Value Chain Governance: Increasing Supplier Competence and Changing Power Relations in the Periphery of Automotive Production—Evidence from Bursa, Turkey. In *European Planning Studies* 19 (1), pp. 77–95. DOI: 10.1080/09654313.2011.530393.

Ponte, S.; Gibbon, P.; (2005): Quality standards, conventions, and the governance of global value chains. *Econ. Soc.* 34 (1), 1–31. DOI: 10.1080/0308514042000329315

Ponte, S.; Sturgeon, T. J.; & Dallas, M. P. (2019): Governance and power in global value chains. In S. Ponte, G. Gereffi, & G. Raj-Reichert (Eds.), Handbook on global value chains (pp. 120–137). Edward Elgar Publishing. DOI: 10.4337/9781788113779

Purnomo, H.; Okarda, B.; Dewayani, A. A.; Ali, M.; Achdiawan, R.; Kartodihardjo, H. et al. (2018): Reducing forest and land fires through good palm oil value chain governance. In *Forest Policy and Economics* 91 (24), pp. 94–106. DOI: 10.1016/j.forpol.2017.12.014.

Quarshie, P. T.; Abdulai, A-R.; Fraser, E. D. G. (2021): Africa's “Seed” Revolution and Value Chain Constraints to Early Generation Seeds Commercialization and Adoption in Ghana. In *Front. Sustain. Food Syst.* 5, p. 9. DOI: 10.3389/fsufs.2021.665297.

Senevirathna, P. (2021): An INGO–corporate partnership in post-war Sri Lanka’s agricultural sector. In *Development in Practice* 31 (7), pp. 961–968. DOI: 10.1080/09614524.2021.1973964.

Serrano, A. (2023): Restructuring palm oil value chain governance in Colombia through long‐term labour control. In *Journal of Agrarian Change* 23 (3), pp. 547–567. DOI: 10.1111/joac.12528.

Sodano, V.; Hingley, M.; Lindgreen, A. (2008): The usefulness of social capital in assessing the welfare effects of private and third‐party certification food safety policy standards. In *British Food Journal* 110 (4/5), pp. 493–513. DOI: 10.1108/00070700810868988.

Sturgeon, T. J.; Memedovic, O.; van Biesebroeck, J.; Gereffi, G. (2009): Globalisation of the automotive industry: main features and trends. In *IJTLID* 2 (1/2), p. 7. DOI: 10.1504/IJTLID.2009.021954.

van der Loos, A.; Langeveld, R.; Hekkert, M.; Negro, S.; Truffer, B. (2022): Developing local industries and global value chains: The case of offshore wind. In *Technological Forecasting and Social Change* 174 (2), p. 121248. DOI: 10.1016/j.techfore.2021.121248.

Wen, H.; Yang, D. Y-R. (2010): The Missing Link between Technological Standards and Value-Chain Governance: The Case of Patent-Distribution Strategies in the Mobile-Communication Industry. In *Environ Plan A* 42 (9), pp. 2109–2130. DOI: 10.1068/a41203.

Xhoxhi, O.; Stefanllari, A.; Skreli, E.; Imami, D. (2020): How intermediaries' power affects contract farming decisions: evidence from the medicinal and aromatic plant sector in Albania. In *JADEE* 10 (5), pp. 529–544. DOI: 10.1108/JADEE-03-2019-0035.

Zhong, D.; Luo, Q.; Chen, W. (2021): Green governance: understanding the greening of a leading business event from the perspective of value chain governance. In *Journal of Sustainable Tourism* 29 (11-12), pp. 1894–1912. DOI: 10.1080/09669582.2020.1864385.

**Additional information 2**

Table 1: Quantitative network survey (adapted from Schusser 2016)

| **Name of actor** | **1: T_q_**  **Information**  0 no or unacceptable  1 good  2 very good | **2: T_v_**  **Info verified**  0 always  1 never  2 sometime | **3: I**  **Incentives**  0 none  1 yes | **4: C_i_**  **Needed Actor**  0 not needed, 1 needed | **5: C_p_**  **Permission**  0 not needed, 1 needed |
| --- | --- | --- | --- | --- | --- |
|  |  |  |  |  |  |
|  |  |  |  |  |  |
|  |  |  |  |  |  |
|  |  |  |  |  |  |

Table 2: Quantitative, qualitative and triangulated results for all three power elements for the direct value chain actors (adapted from Schusser 2016)

| Direct chain actors | Dominant information | | | Incentives | | | Coercion | | |
| --- | --- | --- | --- | --- | --- | --- | --- | --- | --- |
|  | Quantitative | Qualitative | Triangulated | Quantitative | Qualitative | Triangulated | Quantitative | Qualitative | Triangulated |
| Smallholder forest farmers |  |  |  |  |  |  |  |  |  |
| Forest farmers’ organizations |  |  |  |  |  |  |  |  |  |
| Timber traders/ retailers |  |  |  |  |  |  |  |  |  |
| Processing companies |  |  |  |  |  |  |  |  |  |
| Actor *n* |  |  |  |  |  |  |  |  |  |

Quantitative score: 1 if in ‘less powerful group’; 2 if in ‘more powerful group’ (based on the Dominance degree)

Qualitative assessment: + if power source is observed empirically; - if not observed; and 0 if no data

Triangulated score: 1 if power element is ‘not observed’ or ‘no data’, thus in the ‘less powerful group’; 2 if power element is ‘observed empirically’, thus in the ‘more powerful group’

Table 3: Quantitative, qualitative and triangulated results for all three power elements for the coalition members of each direct chain actors (adapted from Schusser 2016)

| Chain actor: smallholders | Dominant information | | | Incentives | | | Coercion | | |
| --- | --- | --- | --- | --- | --- | --- | --- | --- | --- |
|  | Quantitative | Qualitative | Triangulated | Quantitative | Qualitative | Triangulated | Quantitative | Qualitative | Triangulated |
| Spouse |  |  |  |  |  |  |  |  |  |
| Village leader |  |  |  |  |  |  |  |  |  |
| Security guard |  |  |  |  |  |  |  |  |  |
| Intermediary trader |  |  |  |  |  |  |  |  |  |
| Transporter/ harvester |  |  |  |  |  |  |  |  |  |

Quantitative score: 1 if in ‘less powerful group’; 2 if in ‘more powerful group’ (based on the Dominance degree)

Qualitative assessment: + if power source is observed empirically; - if not observed; and 0 if no data

Triangulated score: 1 if power element is ‘not observed’ or ‘no data’, thus in the ‘less powerful group’; 2 if power element is ‘observed empirically’, thus in the ‘more powerful group’
